# THE ROLE OF CAREGIVING IN COGNITIVE FUNCTION AND CHANGE: A REGARDS STUDY

**DOI:** 10.1093/geroni/igac059.2064

**Published:** 2022-12-20

**Authors:** Joanne Elayoubi

**Affiliations:** USF, Land o' Lakes, Florida, United States

## Abstract

Caregiving is often associated with elevated levels of stress and depressed mood. There is some evidence that higher caregiving strain is associated with worse cognitive functioning, however, findings are mixed. The current study examined the relationship between caregiving and cognitive functioning, and the potential mediating effects of depressive symptoms and high-sensitivity C-reactive protein (hsCRP). We identified participants in the Reasons for Geographic And Racial Differences in Stroke (REGARDS) study who were family caregivers at baseline assessment (n=3,118), and then used propensity matching on 14 sociodemographic and health variables to identify 3,118 non-caregivers. Participants completed repeated assessments of global cognitive functioning, learning and memory, and executive functioning and processing speed. Our results showed caregivers and non-caregivers did not differ on cognitive functioning at baseline or in rate of change in cognition over time. Caregivers had higher baseline depressive symptoms than non-caregivers but depressive symptoms and hsCRP did not mediate the relationship between caregiving strain and cognition. The present study, using several methodological advances not found in most previous papers on caregiving and cognition (large sample size, propensity matching of caregivers and non-caregivers, longitudinal methods) found no evidence that caregiving or caregiving strain significantly influenced cognitive performance. Results are consistent with recent research demonstrating that, while caregiving can be highly stressful, many caregivers are resilient and do not show marked declines in cognition and health, or higher mortality. A more balanced narrative of caregiving, emphasizing resilience and both the problems and benefits of caregiving, is indicated.

